# Stallion Sperm Lyophilisation: Effect of Source of Sperm and Reproductive Season

**DOI:** 10.3390/vetsci13080787

**Published:** 2026-08-07

**Authors:** Victoria Luño, Clara Zabalo-Palomo, Maite Olaciregui, Beatriz de la Barreda, Lydia Gil

**Affiliations:** 1 Department of Animal Pathology, Instituto Universitario de Investigación Mixto Agroalimentario de Aragón (IA2), Universidad de Zaragoza, 50013 Zaragoza, Spain; molaciregui@unizar.es (M.O.); lydiagil@unizar.es (L.G.); 2 Laboratory of Equine Reproduction and Equine Spermatology, Veterinary Teaching Hospital, Universidad de Extremadura, 10003 Cáceres, Spain; clarazabalo@unex.es; 3 Centro de Cría Caballar Militar de Zaragoza, Ministerio de Defensa, 50190 Zaragoza, Spain; bdelman@mde.es

**Keywords:** freeze-drying, DNA integrity, epididymal sperm, ejaculated sperm, reproductive season, stallion

## Abstract

This study was conducted to study the quality and DNA integrity of freeze-dried stallion sperm, focusing on the influence of sperm origin (ejaculate vs. epididymis) and seasonality (breeding vs. non-breeding season). Ejaculates were collected from six healthy stallions and epididymal sperm were obtained from 12 stallions via cauda epididymis perfusion during the reproductive season and non-reproductive season in both cases. Sperm samples were diluted in an extender composed of 0.1 M trehalose and BSA 12.5% in PBS, freeze-dried, and stored at 4 °C for at least 3 months. After rehydration, sperm plasma membrane integrity and DNA integrity were analysed using eosin–nigrosin staining and a chromatin dispersion test (SCDt Halomax^®^), respectively. Differences in DNA damage were found to be a function of sperm origin. The percentage of DNA fragmentation in ejaculated sperm was significantly higher than in epididymal sperm. However, when evaluating the effect of the season (breeding vs. non-breeding time), DNA integrity did not differ between seasonal groups. In relation to sperm plasma membrane integrity, no differences were found between the experimental groups. Changes in sperm DNA integrity were observed, independently of season, between epididymal and ejaculated stallion sperm after freeze-drying. This study identifies different factors that lead to improved sperm DNA quality after lyophilisation.

## Simple Summary

Sperm freeze-drying is a promising technique to store and transport genetic samples at room temperature. However, the process can often damage cell viability and DNA integrity. This study investigated whether the source of the sperm (collected via natural ejaculation or directly from epididymis) and the time of year affected the quality of the cell samples. Sperm collected directly from the epididymis had significantly less DNA damage than sperm collected through natural ejaculation. Interestingly, the time of year did not affect these results, and the overall survival rate of the cells remained similar across all groups. These findings are important because they demonstrate that the origin of the sperm is a factor in preserving genetic integrity during freeze-drying. This knowledge will help to develop better preservation methods and support horse breeding program worldwide.

## 1. Introduction

The main sperm preservation techniques are based on decreasing temperature to minimise cell metabolism and maintain its fertile potential. For decades, sperm cooling and freezing methods have been used for this purpose. However, new and innovative preservation strategies such as vitrification and lyophilization have emerged in recent years and are undergoing continuous improvement, offering promising alternatives to traditional methods [1].

Freeze-drying is a preservation method in which ice water under low pressure is removed by sublimation. It has been utilised to preserve viruses, bacteria, yeasts, and fungi for easy storage and transport [2]. Nowadays, freeze-drying is also applied in the preservation of sperm for different mammalian species [3,4,5]. As lyophilised sperm are immotile and unable to fertilise oocytes through in vivo or in vitro methods [6], the use of ICSI (Intracytoplasmic Sperm Injection) for embryo procurement is essential [7]. Lyophilisation offers several advantages, such as the possibility of preserving sperm at room temperature or under refrigeration, which reduces both storage costs and space requirements. It also facilitates sample transportation while lowering associated expenses and minimising the risk of cross-contamination between samples [7,8].

Different factors can affect the quality of freeze-dried sperm samples, including the lyophilisation medium [9]. The main objective of this freeze-dried medium is to preserve the integrity of sperm DNA when cold shocks, reactive oxygen species, and endonuclease activity are encountered during lyophilisation. The most commonly used extender consists of TRIS, HCl, and EGTA [10,11], and includes supplements such as dithiothreitol, diamide, trehalose, sodium (Na^+^ ), and/or potassium (K^+^) ions [12,13,14]. Among all the substances utilized, trehalose allows for water retention, prevents protein denaturation, and inhibits the oxidation of unsaturated fatty acids [15,16]. In addition, it has been shown to revive anhydrobiotic organisms, such as tardigrades, which are exposed to extreme dehydration and rehydration, due to their ability to accumulate trehalose [17]. In sperm, trehalose provides protection against evaporative desiccation processes [6] and improves tolerance against extreme temperatures [18], improving DNA integrity after freeze-drying and increasing the storage time of sperm samples [14,19,20,21]. Furthermore, in somatic cells (lymphocytes and granulosa cells), trehalose increases cell survival after freeze-drying [22,23].

Conversely, sperm quality can be affected by epididymal transit and seminal plasma exposition, with potential impacts on fertilisation and embryo development [24]. Epididymal sperm are more resistant than ejaculated sperm to osmotic stress during conservation in different species, including horses [25,26]. Other factors affecting sperm quality relate to age, genetics, health, management, and environmental exposure or seasonality because horses’ seasonal reproductive activity is modified by photoperiod [27]. Libido, testicular size, and sperm characteristics such as sperm motility, morphology, or DNA integrity can change during the breeding season [28,29]. For this reason, the aim of this study was to determine the effects of reproductive season and cell source (epididymis vs. ejaculate) on stallion sperm DNA integrity after the lyophilisation process.

## 2. Materials and Methods

All chemicals were obtained from Merck (Madrid, Spain) unless otherwise indicated.

### 2.1. Ethics

The study was conducted in accordance with the ARRIVE guidelines, the Spanish Policy for Animal Protection (RD 53/2013), and the European Union Directive 2010/63/EU on animal protection. The protocols were approved by the Ethical Committee for Animal Experiments at the University of Zaragoza (PD10/21NE).

### 2.2. Sample Collection

#### 2.2.1. Epididymal Sperm Recovery

Twenty-four epididymides were collected from 24 different stallions at a local slaughterhouse (Mercazaragoza, Zaragoza, Spain) during the non-breeding (from October to December, *n* = 12) and breeding seasons (from March to May, *n* = 12). Once in the laboratory, one epididymis per stallion were dissected, and blood and extraneous tissues were removed. Sperm were collected by retrograde flushing of the caudal portion of the epididymis using INRA 96^©^ (IMV Technologies, L’Aigle, France) [30]. All epididymal samples showed ≥60% of total motility.

#### 2.2.2. Ejaculated Semen Collection

A total of 24 ejaculates were collected from 6 healthy stallions (4 per animal) during the non-breeding (from October to December, *n* = 12) and breeding seasons (from March to May, *n* = 12) using a Missouri-model artificial vagina. Immediately after collection, semen was diluted 1:1 in pre-warmed (32 °C) INRA 96^®^ extender. The ejaculates used showed a volume greater than 30 mL, concentration of less than 100 × 10^6^ sperm/mL, and a total motility greater than 60%.

### 2.3. Sample Preparation and Freeze-Drying Procedure

All sperm samples were centrifuged at 1000 *g* for 5 min and re-suspended in a freeze-drying solution (Ca^2+/^Mg^2+^-free PBS supplemented with 0.1 M trehalose and 12.5% BSA) [21,23]. Next, 20 µL of each sample (100 × 10^6^ cells/mL) was dropped onto a pre-freezing plastic plate immersed in liquid nitrogen. The spheres of frozen samples were placed into cryotubes (Labcon, Petaluma, California, USA) and then moved to a programmable freeze-dryer (LyoBeta 25, Telstar). Once lyophilised, the vials were vacuum-sealed and stored at 4 °C in the dark at least 3 months.

### 2.4. Rehydration, Sperm Plasma Membrane and DNA Integrity Evaluation

Rehydration was performed by adding 100 µL of Ca^2+/^Mg^2+^-free PBS to each freeze-dried sample. The integrity of the sperm plasma membrane was assessed using eosin–nigrosin staining. The semen sample was mixed 1:1 with a stain solution composed of 5% eosin and 10% nigrosin in citrate solution, and then smeared onto a slide. Eosin penetrated only in dead sperms and stained purple, whereas live sperm appeared white. Nigrosin increased the contrast between the background and sperm cell. A minimum of 500 sperm were counted per semen sample (Olympus BX-40 Olympus U-RFL-T, Tokyo, Japan) at a 400× magnification.

Sperm DNA fragmentation was determined using a sperm chromatin dispersion test designed specifically for equine sperm (Halomax^®^, Halotech DNA SL, Madrid, Spain), in accordance with the manufacturer’s instructions. The agarose was liquefied in a water bath at 95–100 °C for five minutes and then tempered at 37 °C. The sperm sample was added to the agarose and gently mixed. A drop of the sperm suspension was placed on a slide and left in a refrigerator at 4 °C for five minutes. The slide was then immersed horizontally in lysis solution for five minutes, after which it was washed in distilled water. Finally, the samples were dehydrated in two successive baths of ethanol at 70% and 100% concentrations, then air-dried before staining. A commercial green fluorescence staining kit (Fluogreen, Halotech DNA SL, Madrid, Spain) was utilised. The samples were evaluated using fluorescence microscopy (Olympus BX-40, Olympus U-RFL-T, Tokyo, Japan) at 400× magnification. Sperm with intact DNA showed a small, compact halo, whereas sperm with fragmented DNA showed a large halo. At least 500 sperm were analysed in each sample.

### 2.5. Statistical Analysis

The statistical analysis was performed using SPSS version 22.0 for Windows (Chicago, IL, USA). Normality and homoscedasticity of the values was tested. Differences in sperm viability and DNA damage were analysed using a linear mixed model, including the fixed effect of season (breeding vs. non-breeding) and sperm origin (epididymis vs. ejaculate), and stallion identity was specified as a random effect to account for repeated measures on the same animal. The data are expressed as the mean value ± standard deviation (SD), and differences were considered statistically significant at *p* < 0.05.

## 3. Results

The results regarding sperm quality when a linear mixed model was performed for season, sperm origin, and the interaction for each variable are reported in Table 1. There were differences in DNA damage as a function of sperm origin but not the remaining variables or interactions.

**Figure 1:**
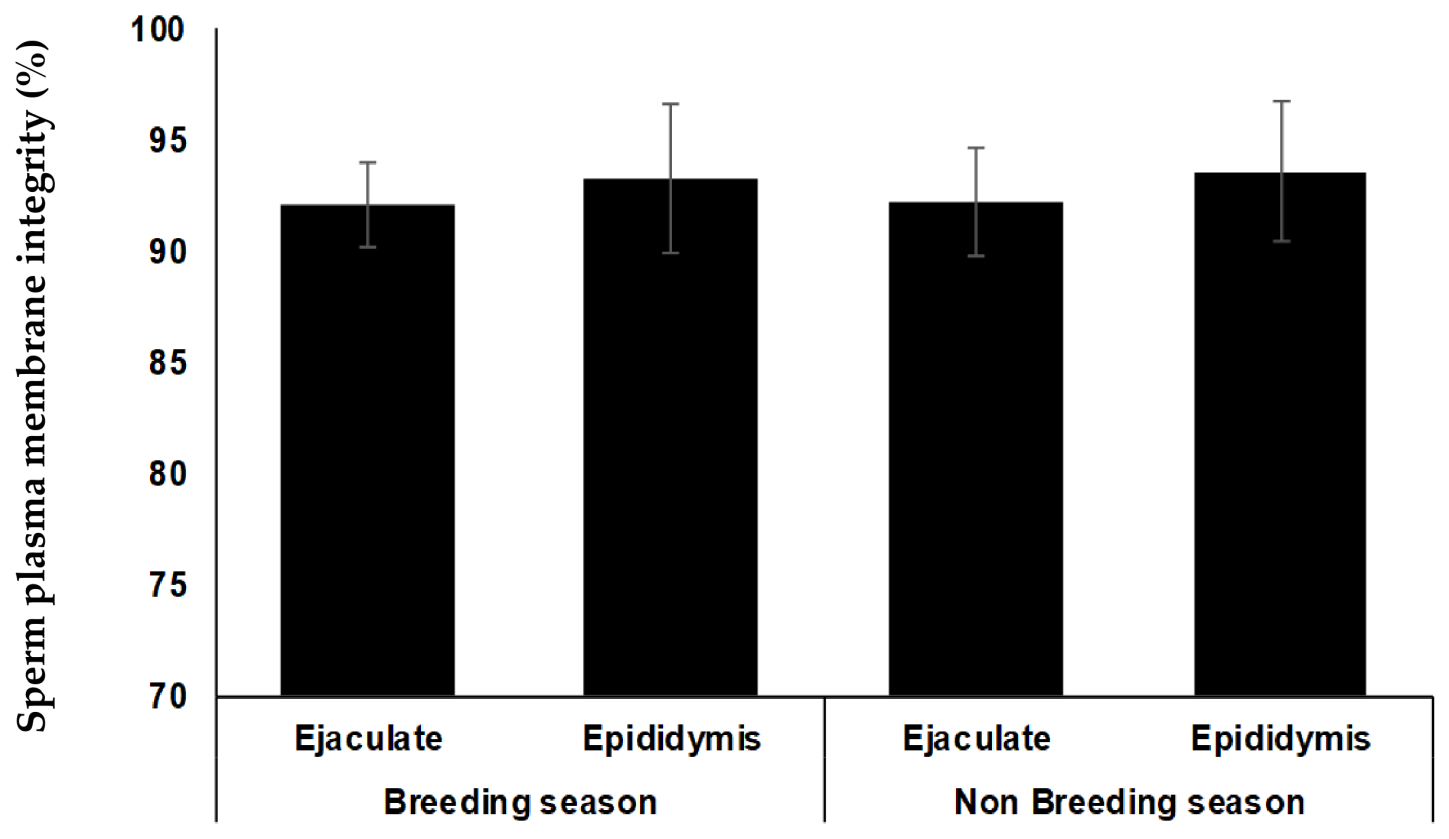
Sperm plasma membrane integrity after freeze-drying in function of season (breeding vs. non-breeding) and cell origin (ejaculate vs. epididymis) (n = 12). Data are presented as mean ± SD.

The proportion of sperm plasma membrane integrity after rehydration is shown in Figure 1. There were no significant differences between the sperm sample groups analysed, with values > 90% of sperm plasma membrane integrity.

**Figure 2:**
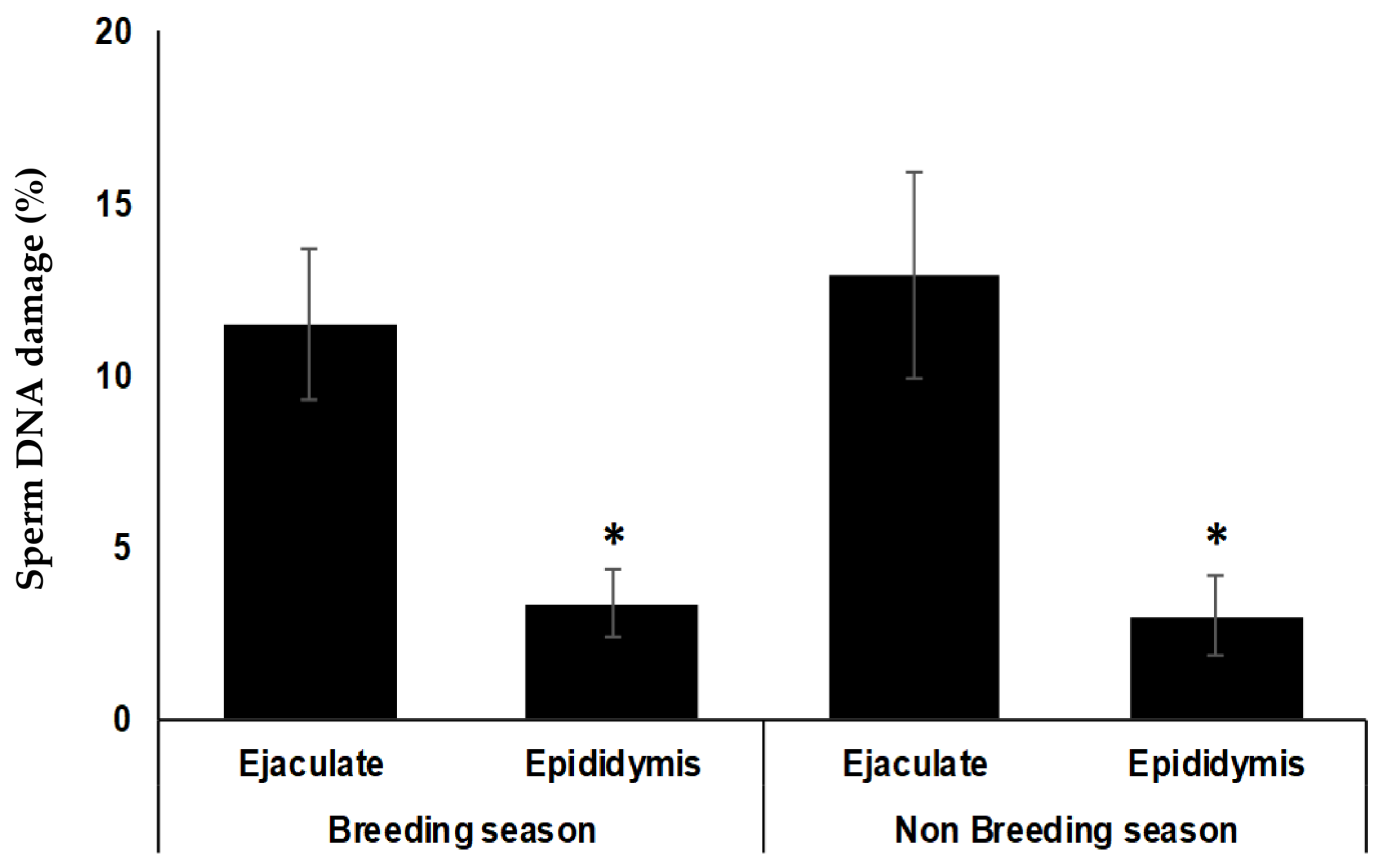
Sperm DNA damage after freeze-drying in function of season (breeding vs. non-breeding) and cell origin (ejaculate vs. epididymis) (n = 12). Data are presented as mean ± SD. * indicates a significant difference in same season (*p* < 0.05).

Figure 2 shows the DNA integrity of the lyophilised stallion sperm. The percentage of sperm DNA damage from ejaculates was significantly higher than those from epididymis (*p* < 0.001), during breeding (11.50% vs. 3.33%) and non-breeding seasons (12.92% vs. 3.00%). However, when the effect of the season was analysed, the results showed that the DNA fragmentation level did not differ between groups.

## 4. Discussion

Freeze-drying is a preservation process that can result in changes to sperm functionality, including total loss of motility, organelle or membrane damage, and alterations to intracellular factors [19]. To minimise these effects, different substances are added to the freeze-drying medium to protect the sperm and maintain its initial characteristics. One of the most common parameters used for determining freeze-dried cell sample quality is plasma membrane integrity [31], as it not only delimits and protects the sperm but also may indicate better preservation of the other cellular structures. In this study, the percentage of sperm plasma membrane damage did not differ between groups, independent of the sperm origin or breeding season. These data could be related to the addition of trehalose to the medium, which confers great stability during sample lyophilisation and storage [32]. Similar result was obtained by [33] when use trehalose in combination to EGCG during freeze-drying of mononuclear cells derived from umbilical cord blood. Vital staining of sperm (such as eosin–nigrosin staining) has several significant technical and clinical limitations. These include subjective visual interpretation, the limited number of cells analysed, and the risk of artefacts. These techniques only measure membrane integrity, but not actual damage to intracellular organelles; therefore, the results must be interpreted with caution.

Although sperm structures can be damaged by lyophilisation, DNA and intercellular factors that activate the oocyte can be preserved. Sperm DNA integrity is vital for obtaining offspring and should be routinely assessed in lyophilised sperm [19]. In this study, we incorporated disaccharides such as trehalose in the preservation medium because they protect DNA sperm during freeze-drying in different species, including horses [14,20,21,34]. When the sample is subjected to drying at high temperatures, trehalose enables the formation of a crystalline matrix that embeds the cell and stabilises it due to the formation of a glassy state that facilitates the mobility of molecules while reducing harmful reactions [35]. Trehalose eliminates the need for chelating agents and offers a simpler alternative for preparing lyophilisation media, as well as provides better results in terms of sperm DNA integrity [14,21]. The incorporation of intracellular trehalose for better cell protection has also been considered, as some studies claim that trehalose is a large molecule and cannot enter the membrane [6]. However, some studies reported that trehalose enters the cells during evaporative drying and provides additional intracellular protection [36].

The percentages of sperm DNA fragmentation determined in this study are lower than those obtained in similar studies with sheep somatic cells, using the same protocol and medium with BSA and trehalose for lyophilisation [23]. This is because sensitivity and the ability to withstand adverse conditions are dependent on each species and cell type [37]. The degree of cellular DNA condensation determines the degree of tolerance to external stimuli, since the less compact the chromatin is, the more susceptible it is to physical and oxidative damage [38]. According to this theory, sperm are characterised by a high level of DNA structuring, which is much more compact than that of other somatic cells [39], and this may be why they better withstand the freeze-drying process.

Sperm recovery from the epididymis is an effective alternative to collecting semen using an artificial vagina. It is performed in specific cases, such as the premature loss of a stallion due to injury or disease or even after castration, allowing the genetics to be preserved [40]. At an experimental level, this technique offers several advantages: easy sample collection, speed, and simple handling. The origin of sperm (ejaculate or cauda epididymis) can affect sample quality, freezing resistance, as well as fertility. Epididymal sperm is more resistant than ejaculated sperm to osmotic stress and cold shock during cryopreservation in different species [25,26,41]. However, the effect of the source of sperm during the freeze-drying procedure has not been tested. In this study, we determined that DNA damage increased in ejaculated sperm compared to epididymal sperm. Different factors can induce DNA degradation during freeze-drying, especially endonuclease activity and oxidative stress [42]. Nuclease activity is present in isolated epididymal and ejaculated sperm, as well as in epididymal fluid and seminal plasma [43]. In some mammalian species, such as mice and humans, nuclease activity has been reported to be greater in ejaculated sperm than in epididymal sperm [43,44,45]. The highest percentages of DNA damage observed in ejaculated freeze-dried sperm in this study could be explained by the variable behavior in function of sperm origin in response to oxidative stress originating from differences in endogenous nuclease activity. These nucleases seem to require Ca^2+^ and/or Mg^2+^ because their activity was inhibited by EDTA/EGTA [10]. However, this inhibition was not complete, suggesting the involvement of some other nucleases with different requirements [43,46,47]. Therefore, the incorporation of EDTA/EGTA into the freeze-dried medium has a limited effect, and other substances that reduce oxidative stress such us trehalose could be more efficient in DNA protection. On the other hand, one limitation of this study was that the ejaculated and epididymal samples did not come from the same animals, and the stallion effect on sperm characteristics could affect DNA integrity results.

In recent years, the effect of breeding season has been tested in relation to sperm DNA damage [48,49,50]. Horses are long-day seasonal breeders, and their sperm characteristics change in relation to the photoperiod [51]. Refs. [29,52] reported that DNA damage in stallion sperm was enhanced during late spring–summer and decreased in spring (breeding season); these data are related to heat stress during spermatogenesis. However, ref. [53] observed that DNA fragmentation increased in December during the non-breeding season, and ref. [48] determined that there were no differences in DNA damage throughout the year in stallion sperm. These results suggest that the individual characteristics of the stallions are responsible for a large proportion of the variability. During cryopreservation, sperm are subjected to oxidative stress via the overproduction of reactive oxygen species, which leads to cell damage and reduces DNA integrity [54]. Seasonal changes (summer–winter) in stallion sperm before and after freezing–thawing have been observed for ROS-related parameters but not chromatin integrity [55]. Similarly, in our study, no differences were found in the percentages of freeze-dried sperm with DNA damage between seasons. However, DNA integrity in frozen–thawed stallion sperm after 3 h of incubation at 37 °C differed between winter and summer samples, indicating that the samples differed in their resistance during incubation depending on season and ROS levels [55]. Determination of ROS-related parameters during lyophilization process could be explain better he results obtained in this study related to sperm DNA damage.

## 5. Conclusions

The evaluation of DNA damage in freeze-dried stallion sperm demonstrated that sperm origin affects DNA integrity, and sperm from ejaculates showed higher levels of DNA fragmentation than epididymal sperm samples. These differences in DNA damage levels were consistently observed regardless of the season of collection, indicating that reproductive seasonality did not affect DNA damage in sperm after freeze-drying. However, sperm samples came from different stallions and the individual effect should be taken into account. The maintenance of sperm plasma membrane integrity post-rehydration suggested that optimisation of the preservation protocol protected the sperm membrane’s structure. These results could help select optimal sperm samples for lyophilisation to create a biobank for equine species.

## Figures and Tables

**Table 1:** Effect of season (breeding vs. non-breeding) and sperm origin (ejaculate vs. epididymis) on DNA damage and plasma membrane integrity.

Variable	DF	DNA Damage		Plasma Membrane Integrity	
		F	*P*	F	*P*
Season	1	0.87	0.357	0.98	0.756
Sperm Origin	1	241.70	<0.001	2.44	0.126
Season * Origin	1	2.26	0.140	0.11	0.918

* = Analysis of the season and sperm origin

## Data Availability

The data presented in this study are available on request from the corresponding author (the data are not publicly available due to privacy).
